# A general review of major global coagulation assays: thrombelastography, thrombin generation test and clot waveform analysis

**DOI:** 10.1186/1477-9560-13-1

**Published:** 2015-01-12

**Authors:** Marcus D Lancé

**Affiliations:** Department of Anesthesiology and Pain Treatment, Maastricht University Medical Center, P. Debyelaan 25, PO box 5800, 6202 AZ Maastricht, The Netherlands

**Keywords:** Global coagulation assays, Thrombosis, Hemorrhage, Hemophilia

## Abstract

Thrombosis and hemorrhage are major contributors to morbidity and mortality. The traditional laboratory tests do not supply enough information to diagnose and treat patients timely and according to their phenotype. Global hemostasis tests might improve this circumstance. The viscoelastic tests (ROTEM/TEG) demonstrated to ameliorate treatment of acute hemorrhage in terms of decreased amount of transfusion and lowered costs. Thrombin generation measurement is indicative for thrombosis and might also become an important tool in managing hemorrhage. While the clot waveform analysis is less well known it could be of worth in staging sepsis patients, early detection of DIC and also in diagnosis and treatment monitoring of hemophiliac patients. Although in different degree all three methods still need more background, standardization and acceptance before a wide clinical application.

## Introduction

Thrombosis and hemorrhage contribute to a large amount of death in terms of ischemic heart disease, stroke and traumatic injuries [1]. For that reason timely diagnosis, risk stratification and monitoring of treatment with anithrombotics or hemostatic agents becomes crucial. The traditional coagulation tests (aPTT and PT/INR) have been developed while discovering the coagulation cascade and contributed to our current understanding. The tests have been standardized for monitoring therapy (i.e. vitamin K antagonist-INR and heparin-aPTT). Moreover they have a longstanding position in diagnosis and management of factor deficiencies including hemophilia and are licensed by many authorities (e.g. FDA) for this purpose. Although they are recommend for management of acute (acquired) hemorrhage these tests need some laboratory turnaround time and may not reflect the complexity of the hemostatic impairment. In this light the traditional coagulation tests (aPTT and PT/INR) are scrutinized for this clinical applicability. On one hand these tests never were developed nor were evaluated for predicting bleeding risk and treatment of acute bleeding patients [2, 3]. That is why current guidelines consider the use of global hemostatic coagulation tests in management acute hemorrhage [4, 5]. On the other hand the reporting of these assays mostly stops at the moment the so-called thrombin burst starts. That is to say the traditional tests inform on the initiation of clotting but not the hemostatic capacity in terms of clot formation and maximal thrombin generation although this is technical possible (e.g. clot waveform analysis).

Yet, since the early phase of coagulation research there were reports on more global assays, which nowadays gain more attention. In 1948 Hartert presented the first viscoelastic test, which he called thromboelastography [6]. Little later in 1953 it was MacFarlane and Biggs who reported a measurement of thrombingeneration in blood [7]. However at that moment both techniques were very time and work intensive which made them not applicable in clinical practice.

An ideal coagulation test should be easily to perform and quickly to obtain while giving high reliable and robust results. The assay should allow an accurate estimation of the thrombotic risk and the risk of bleeding. Furthermore it should employ flow conditions, endothelial interaction, platelet contribution as well as physiologic conditions such as pH and temperature. In short such an ideal coagulation test currently does not exist. However there are attempts to develop assays, which fulfill these requests, at least partially.

The present review describes the viscoelastic tests (TEG/ROTEM), thrombin generation test (TGT) and the clot waveform analysis (CWA) as global clotting test giving some background information, clinical applications and finally information on their limits.

## Viscoelatic tests (thromboelastography, thromboelastometry)

Hartert presented in 1948 a method to monitor the dynamics of the complete coagulation process in whole blood, which he called thromboelastography [6]. In the original method fresh whole blood was triggered with a coagulation activator of the contact pathway (celite) and put into a cup. Then a torsion wire was brought into the mixing cup, which was continuously turned with an angle of 4^∘^45’. As the gradual formation of the clot strength increases the movement of the torsion wire damps until it becomes almost fixed in the moment of maximal clot stability. Tracings of the movement over time reflect the characteristic graph, from which the start of clot formation, and maximal stability of the clot can be read (Table 1). The method also detects gradual resolution of the clot due to fibrinolysis. Soon after its description, this viscoelastic method was criticized to be too global. Another criticism was its high sensitivity to external vibrations and the lack of detecting single factor deficiencies [6, 8, 9]. Moreover the test was time and work intensive, which discouraged a wide spreading of the method. After introducing improvements to allow automation of the technique and implementation of a myriad of trigger reagents, which give information on the extrinsic pathway (tissue factor), the effects of the fibrin fraction on clot forming (platelet inhibitor-cytochalasin D/abciximab) but also on specific questions such as heparin effect (heparinase test) or resistance to lysis (aprotinin test) thrombelastography has become a method with a wider range of applications [10]. In the mid 1980ties, the method was picked up as a potential bedside point-of-care test for monitoring hemostasis during liver transplantation and cardiac surgery [11, 12].

**Table 1:** **Thromboelastography parameters**

Variable	TEG	ROTEM
From start until 2 mm baseline	R	Clotting time (CT)
From 2–20 mm above baseline	K	Clot formation time (CFT)
Alpha angle (°)	Slope	Angle of tangent at 2 mm
	Between R&K	
Maximum strength	Maximal amplitude	Maximal clot firmness
	(MA)	(MCF)
Clot lysis (CL) at minutes	CL 30, CL 60	LY30, LY 60

Currently, two (semi)automated commercial devices are on the market. The thromboelastometry (ROTEM-analyzer, TEM international, Muenchen, Germany) instrument uses a fixed cup with a pin that is rotating. The other thrombelastography (TEG-analyzer, Haemonetcis Corp., Braintree, MA, United States) system makes use of the classical method with rotating cup [13]. While being designed as kinetic tests, either method effectively measures the capacity of the coagulation process in terms of maximal fibrin clot formation [14]. During the last years, these tests have been shown to be suitable for detecting and treating coagulopathy in trauma care, cardiac surgery and liver transplantation, particularly in patients were fibrinogen levels are low [15–17]. Thromboelastography seems to become an important tool in detecting coagulopathies and guiding hemostatic therapy at the bedside, especially in the operation theater [4]. It has been shown to detect excess heparin effect, but also to catch signs of hemodilution in cardiac surgery. Treatment upon these results could be shown to save transfusion of blood products and reduce costs [18]. As a point of care device it may also monitor hemorrhage and control therapy during obstetric surgery, at the emergency department, and at the intensive care unit [13, 19]. Recently, there are reports on the efficacy of the device when used in pediatric craniosynothesis surgery. Here the need for transfusion of fresh frozen plasma and platelet transfusion could be dramatically reduced [20]. Although the thromboelastography is able to detect hypercoagulable situations it is not frequently used to predict thrombosis rather to tailor antithrombotic therapy. This might also be due to the fact that thromboelastography does not reflect the effects of LMWH’s and (new) oral anticoagulants completely.

As in any assay there are some blind spots in this method. Platelet dysfunction either inherited or drug induced will not be detected. Another shortcoming is the insensitivity to detect the effects of von Willebrand Factor, which is involved in the initiation of clot forming. Finally, factor XIII, which is mainly responsible for stabilization of the fibrinogen network, is also not adequately displayed [21, 22]. Some of these shortcomings might be overcome by spiking samples with platelet activators or specific antibodies [22]. Finally there are still some concerns about standardization of the assays. Recently an investigation on quality control and assurance showed a wide variation of TEM results between different centers when comparing plasma sample analysis. Hereupon a working group was set up to homogenize the variability of this test [23, 24]. However, the variability in whole blood seems acceptable for the purpose of bleeding management [25, 26].

## Thrombin generation

MacFarlane and Biggs described in 1953 the thrombin measurement in whole blood [7]. In the same issue of the journal Pitney and Dacie did so in plasma [27]. Although the authors could identify hemophiliac patients the test was very work and time intensive, because the measurement had to be done by continuously subsampling. To overcome this problem many years later Hemker and Beguin developed a method using a chromogenic substrate. Unfortunately the selected substrate exhausted quickly and did not reflect the complete amount of thrombin generating. Moreover it inhibited the physiological feedback loops of thrombin so it interfered strongly with the measurement. Hemker improved the assay by changing to another substrate (MeO-mal-Aib-Arg-pNA). However the reaction product of this substrate had to be determined by optical measures that only could be performed in defibrinated plasma. Shortly hereafter the same group replaced the chromogenic substrate by a fluoroscopic agent (Z-Gly-Gly-Arg), which was bound to 7-amino-4-methylcoumarin. With this substrate the read out could be done automatically by monitoring the fluorescence signal. By comparing it with a calibrator of known thrombin activity the development of the calibrated automated thrombin generation (CAT) was possible [28]. Next to this system there are several thrombin generation tests commercially available, which rely on fluorogenic or chromogenic principles (manual and automated fluorogenic assay by Technclone, chromogenic assay by Dade Behring and customized tests such as the Novel Hemostasis Assay from the Radboud University Medical Centre, Nijmegen, The Netherlands).

In general thrombin generation tests (TGT) uses some trigger to imitate vessel wall damage (e.g. tissue factor). In platelet poor plasma (PPP) procoagulant phospholipids (in general about 4 μM) amplify the effects of tissue factor [29]. Depending on the question of the test the amount of added TF may reflect different kinds of factor compositions. That means with large amounts (>10pM) TF factor VIII, IX and XI are bypassed, but between 2 and 5 pM TG depends on factor VIII and IX and at even lower concentrations factor XI might become more important [30]. In contrast when using platelet rich plasma (PRP) the platelets take the role of phospholipids over as amplifying surface. By this the reaction reflects the interplay of platelet activation and plasmatic coagulation. The most relevant parameters deriving from the thrombin generation tests are the lag-time (time to start), the time to peak, the peak height, and the endogenous thrombin potential (ETP) [28].

After its initial use as a research tool the TGT shows increased thrombin generation in thrombophilic states such as venous thrombosis due to deficiency (e.g. AT, protein C or S, deficiency) as well as due to APC-resistance and the antiphospholipid syndrome [31, 32]. However this depends on activator and additives (e.g. thrombomodulin, activated protein C). Moreover is increased thrombin generation associated with arterial thrombosis such as ischemic stroke, but also with acute coronary syndromes [33, 34]. In these scenarios assessment of TG could be helpful guiding therapy with antithrombotics while trying to avoiding bleeding. Finally thrombin generation gives important information in hemorrhagic disease either inherited (hemophila A and B) or acquired (factor deficiency, VKA-therapy) [33, 35]. In hemophiliac patients the TGT might describe the bleeding tendency and so the risk of bleeding better than the traditional tests. Particularly the bypassing therapy of hemophiliacs with inhibitors might improve, but this is still matter of debate [36–39].

When compared to the viscoelastic tests, which asses the fibrin clot formation in general, the TGT could give more information on the hemostatic capacity in total, because the generation of thrombin does not stop at the moment when the fibrin clot has been generated [28]. In a recent clinical study it could be shown that TGT gives additive information managing bleeding patients [40]. Bosch et al. recently demonstrated the additive value of the TGT when assessing patients undergoing cardiac surgery. The authors could show that the TGT was able to predict bleeding [41].

However the TGT is performed in platelet poor plasma (PPP) and/or platelet rich plasma (PRP), which needs time for preparing and makes this method not suitable for quick diagnosis. A more recent development is the whole blood thrombin generation test, which allows the presence of erythrocytes and other blood cells. This may be an advantage because the blood cells contribute to coagulation in vivo, which may be underestimated in plasma TG. Additionally using whole blood saves time because the step of spinning the blood is not needed. This might accelerate the analysis and makes this test suitable as a bedside test. However this is still a matter of research [42].

Of course there are some disadvantages of the TGT, too. Major drawback was the duration of the test (e.g. CAT), which makes it not suitable for emergency cases. On the other hand the assay is not enough standardized for broad clinical use, which still hampers its approval as routine clinical tool. There is large variance due to preanalytic variables and there is a lack of reference ranges for the specific conditions (kind and amount of trigger substances) [43, 44]. Still there is debate about the need to use inhibitors of the contact activation pathway (e.g. corn trypsin inhibitor), because a factor XII pathway activation due to sample tube contact might interfere with the results [45].

## Clot waveform analysis (CWA)

Principally clot waveform analysis (CWA) is based on the traditional aPTT assay. It is a technique reported by Braun and co-workers who assessed the aPTT and PT with light transmission [46]. Yet, the registration is photo-optically and the read out is prolonged, which creates a graph registered over time instead of the clotting time known from the aPTT. The graph is computerized and the first and second derivatives are added to the final plot. The tracing against the time should reflect the whole process of clot formation and clot lysis. For better standardization it seems important that the assay needs some specific reagents, which do not interfere with light transmission/absorption [47].

The parameters are given as the transmittance trace, its first derivative, which gives information on the velocity of coagulation and its second derivative, which informs about the acceleration and deceleration of coagulation. In each of the graphs three phases are distinguished: a pre-coagulation phase, a coagulation phase and a post-coagulation phase with set of ten parameters each [48]. Although the method still needs standardization there are a couple of possible clinical applications.

In the first instance this test was used to monitor the course of disseminated intravascular coagulation, a disease, which frequently was seen in critically ill patients. The findings were independently from standard aPTT measurements. Using this test as a global assessment tool a DIC might be diagnosed with a high specificity (97.6%) and sensitivity (98%) [49, 50]. Moreover this assay could even detect the DIC earlier than conventional methods in up to 19% of the cases. For that reason the test is recommended by the guidelines for diagnosis and treatment of DIC [51].

Besides these findings the test seems sensitive to even mild factor deficiencies (FXII, X, IX, VII, V and II). In this light the assay gives information on hemophiliacs. It might help to distinguish between hemophilia A and B. Moreover the assay gives information on the clinical phenotype with regard of bleeding tendency. Doing so it might help monitoring treatment of these patients with factor concentrates but also with bypassing agents [52].

Some authors challenged the test in critically ill patients suffering from sepsis. Here they could show that the severity and the prognosis of sepsis may be predicted by the CWA [53, 54]. The CWA results were more accurate than standard inflammation parameters (C-reactive protein and procalcitonin) [55]. These findings were recently confirmed in a pediatric population suffering from meningococcal infection [56].

Although the CWA is inexpensive and easily to perform there is some disadvantage impeding its widely use. Currently there are only two systems, which are able to assess the light transmittance or absorbance tracings. However analyzers, which work with the same principles should be able to create the graphs after updating with the necessary software. Another drawback is the obligation to use clear reagents, which do not interfere with the light beam. This might also form a problem in case of colored plasma (hyperbilirubinemia, hyperlipidemia or hemolysis) [47]. Due to the fact that there is not much experience with this assay the parameters seem quite unknown and there is not much literature with regard to clinical validation.

## Conclusions

Our traditional coagulation tests do not cover all information the clinician needs to diagnose and treat thrombophilia, hemorrhage and inherited coagulation disorders. Global coagulation assays such as viscoelastic tests (TEM/TEG), thrombin generation test and clot waveform analysis care several advantages. While the viscoelastic tests proved to be worthwhile in management of acute hemorrhage, the thrombin generation test showed to be of use in thrombosis (venous and arterial) but also it might be a meaningful instrument in hemostatic therapy. The latter technique is at the beginning of a broad clinical use. Clot waveform analysis is even less well known. Although there is reasonable suspicion that this method might improve diagnosis and treatment of DIC, sepsis and hemophilia its application is not wide spread. Yet, there is need for more clinical data to support the current evidence.

## Author contribution

MDL is responsible for writing and editing of the manuscript.

## Abbreviations

aPTT: Activated partial thromboplastin time
CAT: Calibrated automated thrombin generation
CWA: Clot waveform analysis
DIC: Disseminated intravascular coagulation
ETP: Endogenous thrombin potential
INR: International normalized ratio
LMWH: Low weight molecular heparin
PPP: Platelet poor plasma
PRP: Platelet rich plasma
PT: Prothrombin time
TF: Tissue factor
TG: Thrombin generation
TGT: Thrombin generation test
TM: Thrombomodulin
VKA: Vitamin K antagonist.
